# Altered abundances of human immunoglobulin M and immunoglobulin G subclasses in Alzheimer’s disease frontal cortex

**DOI:** 10.1038/s41598-022-10793-w

**Published:** 2022-04-28

**Authors:** Rukmani Lekhraj, Shirin Lalezari, Jennifer T. Aguilan, Jiyue Qin, Simone Sidoli, Wenzhu Mowrey, Seema Gollamudi, Parviz Lalezari

**Affiliations:** 1 Neurological Surgery Research Laboratory, Department of Neurosurgery, Montefiore Medical Center and Albert Einstein College of Medicine, Bronx, NY USA; 2 Department of Pathology, Albert Einstein College of Medicine, Bronx, NY USA; 3 Department of Epidemiology & Population Health, Albert Einstein College of Medicine, Bronx, NY USA; 4 Department of Biochemistry, Albert Einstein College of Medicine, Bronx, NY USA

**Keywords:** Alzheimer's disease, Neuroimmunology

## Abstract

The immune system has been described to play a role in the development of Alzheimer’s disease (AD), but the distribution of immunoglobulins and their subclasses in brain tissue has not been explored. In this study, examination of pathologically diagnosed frontal cortex gray matter revealed significantly higher levels of IgM and IgG in late-stage AD (Braak and Braak stages V and VI) compared to age-matched controls. While levels of IgG2 and IgG4 constant region fragments were higher in late-stage AD, concentration of native–state IgG4 with free Fc regions was increased in AD III and VI. RNA analysis did not support parenchymal B-cell production of IgG4 in AD III and V, indicating possible peripheral or meningeal B-cell involvement. Changes in the profile of IgM, IgG and IgG subclasses in AD frontal cortex may provide insight into understanding disease pathogenesis and progression.

## Introduction

Alzheimer’s disease (AD) is a neurodegenerative disorder that is characterized by the abnormal accumulation of beta amyloid (Aβ) peptides and phosphorylated Tau (p-Tau) proteins^1–6^. While dysfunction of neuroimmune cells has been associated with protein aggregation and inflammation, it is not the sole immunopathology in AD^7–11^. The involvement of B-cells in AD pathogenesis has been extensively investigated and has yielded conflicting results. Depletion of B-cells has been associated with improved cognition and decreased amyloid deposits or accelerated disease progression^12,13^. These contradictory findings may be attributed to the mouse model used or the immunoglobulins (Ig’s) that are produced by B-cells. Ig’s were found to co-localize with degenerating neurons in AD parenchyma, suggesting that they play a larger role in neurodegeneration^14^. IgM antibodies against Aß were demonstrated to be reduced in the plasma of patients with AD^15^. IgG’s have been shown in various mouse models to cross the blood brain barrier and promote clearance of Aβ^13,16^. Intrathecal synthesis of IgG was shown to be positively correlated with medial temporal lobe atrophy in AD, but the examination of IgG subclasses in the parenchyma have yet to be explored^17^.

IgG subclasses, IgG1, IgG2, IgG3, and IgG4_,_ are termed according to their prevalence in blood and have different functional roles with respect to their target antigen^18^. IgG1, IgG2, and IgG3 have the ability to fix complement, which facilitates disintegration of their targets and promotes phagocytosis. IgG4 has a high affinity Fab region, but due to its low affinity Fc region it does not possess the ability to fix complement^18,19^. These different biological activities enable each subclass to protect against infection, while dysregulated production of any IgG subclass can result in disease manifestation^18,20–22^. In certain autoimmune diseases, independently altered serum levels of IgG1, IgG2, and IgG3 were demonstrated to play a pathogenic role in autoimmunity, whereas IgG4 was not considered a pathogenic player in these diseases due its inability to cause complement mediated tissue injury^23,24^. In IgG4-autoimmune diseases, elevated levels of antigen-specific IgG4 autoantibodies bind to its targets and inhibits their function, resulting in disease pathogenesis^24^. While the specific role that these subclasses may play in AD is unknown, recent studies have yielded some insight. Intrathecal synthesis of IgG3 and IgG4 was demonstrated in patients with moderate AD, while advanced stages exhibited production of all subclasses^25^. Moreover, it has been shown that brain tissue of individuals with neurodegenerative diseases exhibit increased expression of Fc gamma receptors (FcγR), which promotes neuroinflammation and increases neurodegeneration^26–28^. The varying specificities and affinities of FcγRs for IgG subclasses have been studied to highlight possible therapeutic and pathogenic effects of antibodies in disease^29^. Thus, investigating the production and distribution of Ig isotypes and subclasses in the brain parenchyma may improve our understanding of the pathogenic mechanism underlying AD.

In this study, the levels of immunoglobulins (IgM, IgG and IgG1-4) were measured in fresh-frozen frontal cortex gray matter from pathologically diagnosed AD (Braak and Braak stages III, V and VI) and age-matched cognitively normal (Normal) individuals. We found altered Ig profiles in AD parenchyma compared to Normal. Elevated levels of IgM, IgG4 and IgG2 were seen predominantly in AD V and AD VI. IgG4 levels were elevated in AD III. Taken together, the significant differences in brain Ig levels supports a relationship between the humoral immune response and AD progression.

## Results

### Tissue samples

A total of 25 postmortem cortical tissue samples from individuals that were pathologically diagnosed with varying Braak and Braak stages III, V, and VI of AD, together with age-matched cognitively Normal controls (Normal) were obtained from New South Wales (NSW) and Kathleen Price Bryan Brain Bank at Duke University (Duke). The age, sex, postmortem delay (PMD), neuropathological diagnosis, cause of death, and APOE genotype—if available—are listed for each sample in Supplementary Table 1. Tissue samples consist of nine Normal, five AD III, six AD V, and five AD VI.

### IgG and IgM levels are different between late-stage AD and normal subjects

**Figure 1:**
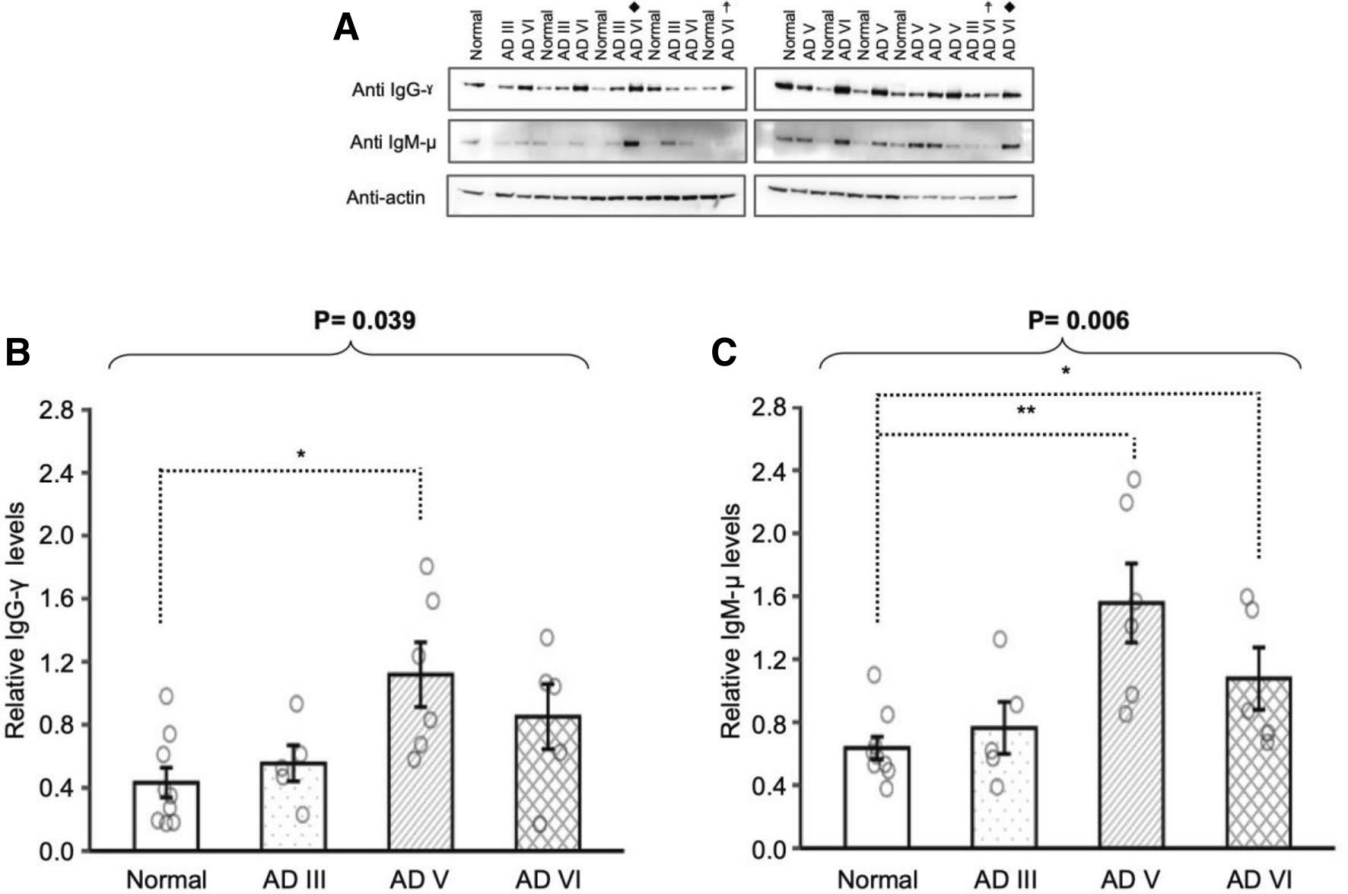
Western blot analyses of human IgG gamma chain and human IgM mu chain were performed on denatured soluble proteins from cortical tissue supernatants of age-matched cognitively normal (Normal), AD III, AD V and AD VI subjects, with two samples duplicated on both membrane for normalization between membrane, denoted with filled rhombus and ☨, is presented as cropped band of interest (**A**). Compared to Normal, human IgG-γ levels were increased in AD V (**B**) and human IgM-µ levels were increased in both AD V and AD VI (**C**). Data was normalized using HRP-conjugated Beta-Actin monoclonal antibody and presented as mean ± SEM. Overall difference was determined with Kruskal–Wallis test and pairwise comparisons were conducted with Wilcoxon rank sum tests. *p ≤ 0.05, **p ≤ 0.01 and ***p ≤ 0.001.

To investigate the levels of IgM and IgG in different stages of AD brain tissue, supernatants produced from homogenized cortical gray matter were subjected to western blot analysis using anti-human antibodies for heavy chains Gamma (IgG-γ, Kruskal–Wallis test: p = 0.039) and Mu (IgM-µ, Kruskal–Wallis test: p = 0.006) (Fig. 1). The level of IgG-γ (Fig. 1A) was significantly higher in AD V compared to Normal (p = 0.012). Similarly, the level of IgM-µ was significantly higher in late-stage AD (AD V, p = 0.002 and AD VI, p = 0.019) compared to Normal.

### IgG subclasses in AD differ from normal subjects

**Figure 2:**
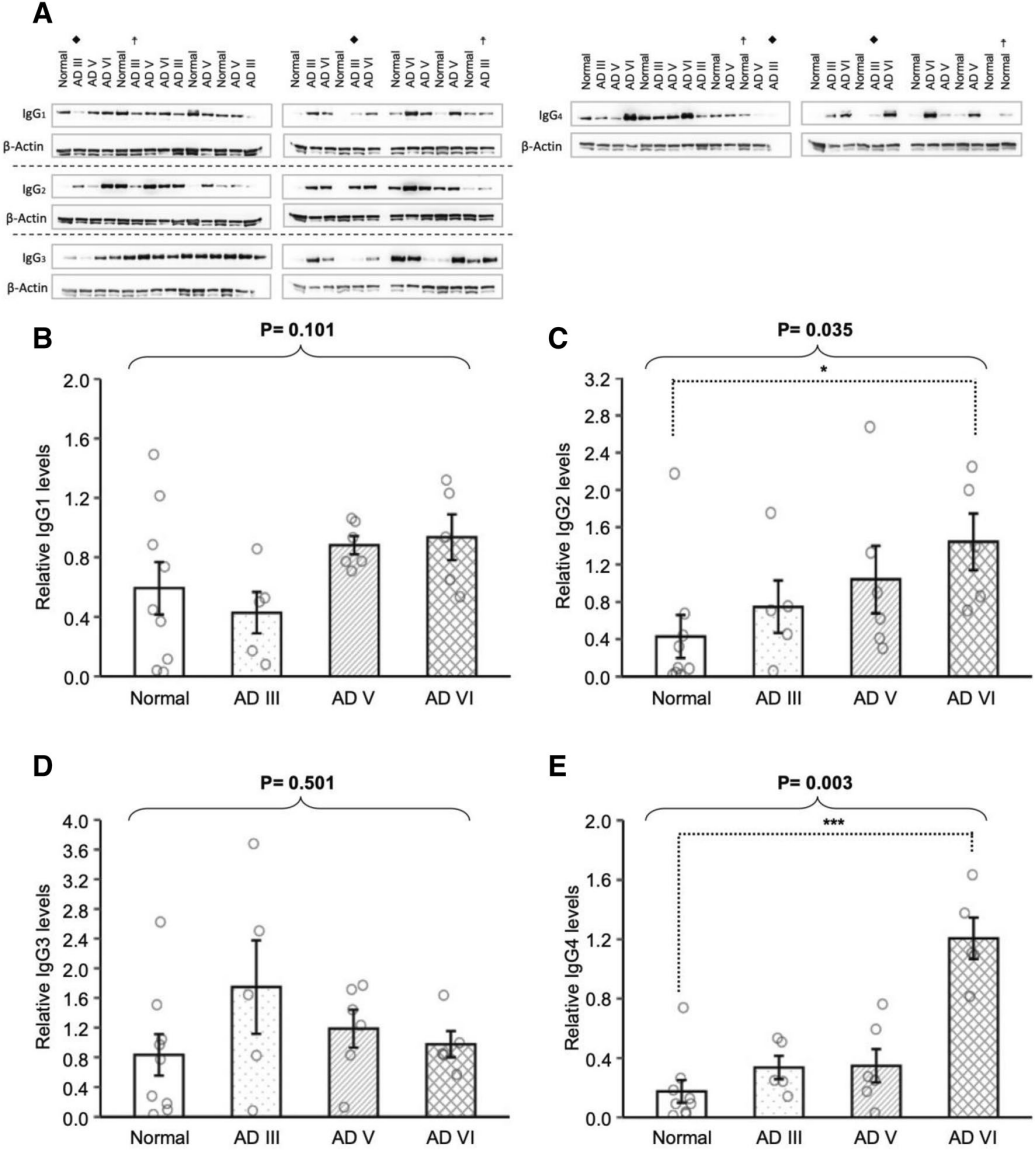
Denatured soluble proteins from cognitively normal (Normal), AD III, AD V and AD VI cortical tissue supernatants were subjected to western blot analysis and probed with anti-human IgG subclass specific antibodies targeting each hinge region: IgG1 (**B**), IgG2 (**C**), IgG3 (**D**) and IgG4 (**E**). Each experiment, presented as cropped band of interest, included two samples that were duplicated on both PVDF membranes as basis for normalization across membranes, denoted with filled rhombus and ☨ (**A**). IgG2 (**C**) and IgG4 (**E**) levels were increased in AD VI. Data was normalized with HRP-conjugated Beta-Actin monoclonal antibody upper band and presented as mean ± SEM. Overall difference was determined with Kruskal–Wallis test and pairwise comparisons were conducted with Wilcoxon rank sum tests. *p ≤ 0.05, **p ≤ 0.01 and ***p ≤ 0.001.

The 4 subclasses of human IgG were evaluated to further refine whether the IgG-γ elevation seen among late-stage AD samples could be subclass dependent. Samples were evaluated for the protein expression of each IgG subclass, IgG1, IgG2, IgG3, and IgG4, using antibodies targeting the hinge region of the Ig (Kruskal–Wallis test: p = 0.101, p = 0.035, p = 0.501, and p = 0.003, respectively) (Fig. 2A–D). There was a significant increase in the levels of IgG2 (p = 0.012) and IgG4 (p < 0.001) in AD VI compared to Normal.

**Figure 3:**
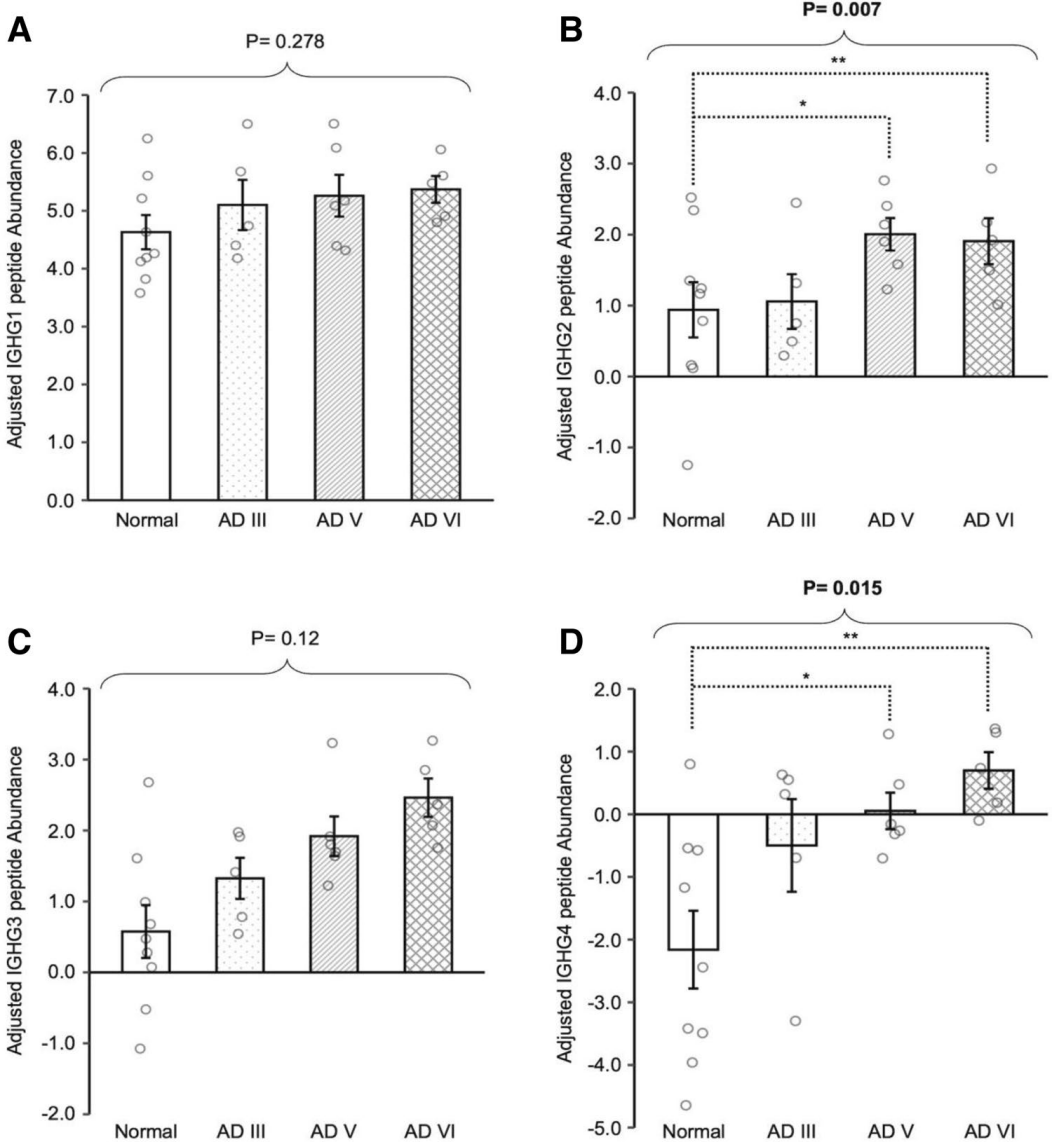
Proteomic analysis on soluble proteins from cortical tissue supernatants of cognitively normal (Normal), AD III, AD V and AD VI subjects evaluated peptide abundance from each IgG subclass: IGHG1 (**A**), IGHG2 (**B**), IGHG3 (**C**) and IGHG4 (**D**). Adjusted peptide abundance of IGHG2 (**B**) and IGHG4 (**D**) were higher in AD V and AD VI compared to Normal. Adjusted peptide abundance is presented as mean ± SEM. Overall difference was determined with Kruskal–Wallis test and pairwise comparisons were conducted with Wilcoxon rank sum tests. *p ≤ 0.05, **p ≤ 0.01 and ***p ≤ 0.001.

Proteomic analysis performed on brain supernatants demonstrated that the levels of peptide abundance among the IgG subclasses, IGHG1, IGHG2, IGHG3, and IGHG4, were individually altered in each pathological stage of AD (Kruskal–Wallis test: p = 0.278, p = 0.007, p = 0.120, and p = 0.015, respectively) (Fig. 3A–D). The levels of IGHG2 and IGHG4 were demonstrated to be significantly elevated in AD V (p = 0.012 and p = 0.018) and AD VI (p = 0.007 and p = 0.007) compared to Normal.

Detection of Ig heavy chain constant region RNA expression using RT-qPCR are presented as fold changes in Table 1. Percentages were calculated based on positive detection of RNA expression for each condition. In the Normal group (n = 9), 77.7% of subjects expressed both IGHG1 and IGHG3, 11.11% expressed IGHG2, and 22.22% expressed IGHG4. In AD III (n = 4), 75.00% of subjects expressed IGHG1, 50.00% expressed IGHG2, 100.00% expressed IGHG3, and 25.00% expressed IGHG4. In AD V (n = 6), 50.00% of subjects expressed IGHG1, 50.00% expressed IGHG2, 66.67% expressed IGHG3, and 16.67% expressed IGHG4. In AD VI (n = 5), 60.00% of subjects expressed IGHG1, 80.00% expressed IGHG2, 100.00% expressed IGHG3, and 60.00% expressed IGHG4.

**Table 1:** RNA extracted from cognitively normal (Normal), AD III, AD V and AD VI cortical tissue samples were subjected to RT-qPCR to detect IGHG1, IGHG2, IGHG3 and IGHG4 RNA specific to each IgG isotype hinge region.

Sample ID	Condition	IGHG1	IGHG2	IGHG3	IGHG4
787	Normal	0.24	4.25	1.13	Undetermined
1035	Normal	0.21	Undetermined	Undetermined	Undetermined
1169	Normal	1.00	Undetermined	1.09	Undetermined
1600	Normal	7.49	Undetermined	Undetermined	Undetermined
1609	Normal	0.35	Undetermined	1.04	16.71
OO	Normal	Undetermined	Undetermined	555.46	Undetermined
VV	Normal	55,731.38	Undetermined	188,535.38	Undetermined
AAA	Normal	2.03	Undetermined	3.21	12.16
CCC	Normal	Undetermined	Undetermined	708.94	Undetermined
645	AD III	0.47	2.85	0.29	9.06
763	AD III	7.00	Undetermined	18.68	Undetermined
805	AD III	1.06	Undetermined	0.67	Undetermined
867	AD III	Undetermined	16.48	3.96	Undetermined
PP	AD V	Undetermined	Undetermined	1520.52	Undetermined
QQ	AD V	Undetermined	100.22	Undetermined	Undetermined
WW	AD V	0.64	Undetermined	0.93	Undetermined
UU	AD V	Undetermined	Undetermined	21.19	Undetermined
BBB	AD V	9.49	24.97	90.55	Undetermined
846	AD V	0.22	1.58	Undetermined	2.33
969	AD VI	Undetermined	2,137,684.42	0.65	Undetermined
1068	AD VI	4.65	8.84	4.33	23.44
1200	AD VI	33.87	30.69	3.94	191.86
1547	AD VI	4.29	11.60	1.21	98.56
DDD	AD VI	Undetermined	Undetermined	29,178.68	Undetermined

After normalization using eukaryotic 18S rRNA, detection frequency is presented as fold changes using 2^−ΔΔCT^ method.

### Concentrations of native-state IgM and IgG1-4 are altered in various stages of AD

**Figure 4:**
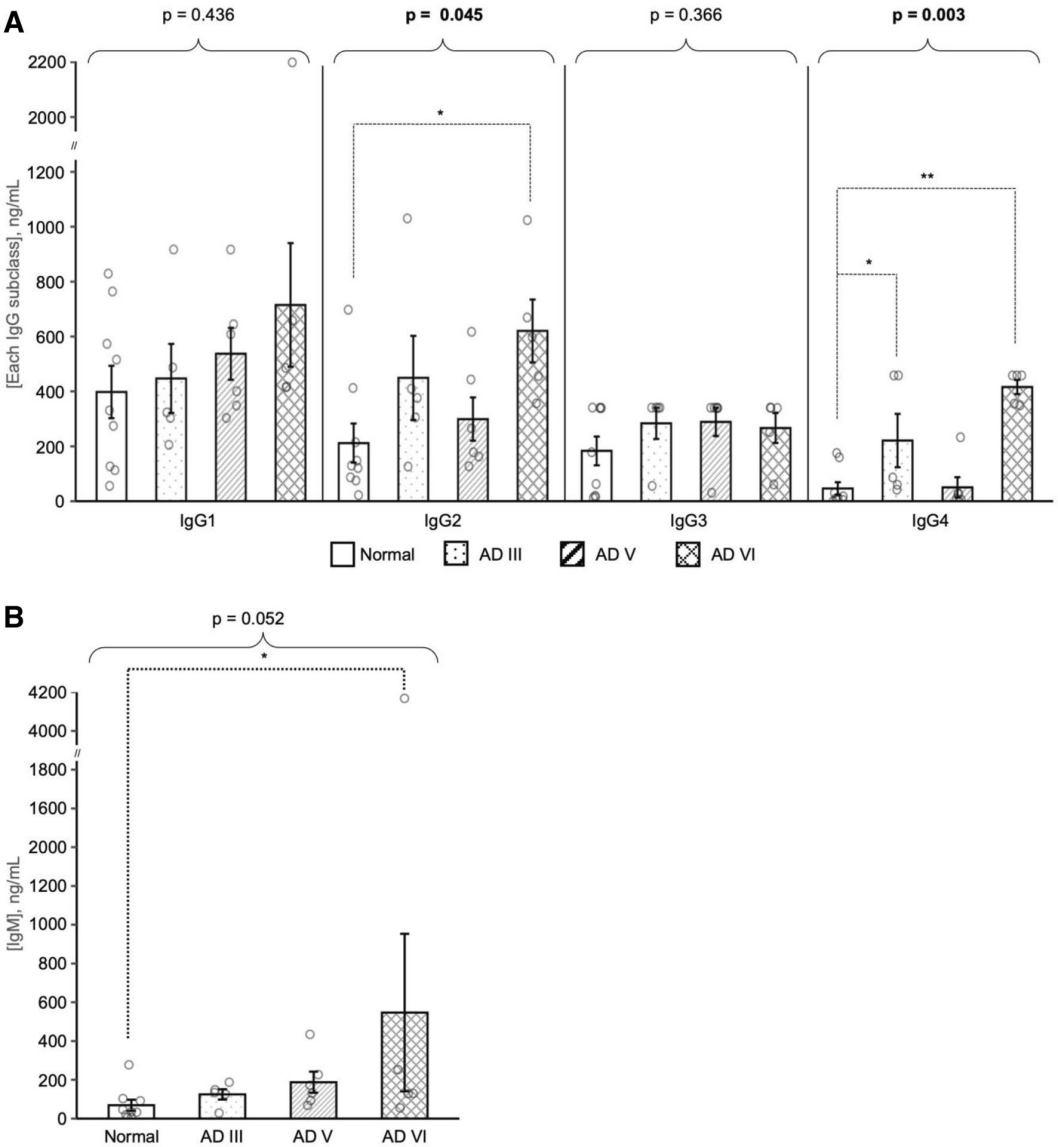
Native cortical tissue supernatants from age-matched cognitively Normal, AD III, AD V and AD VI subjects were quantified using the Human Immunoglobulin Isotyping Multiplex Assay for IgG1, IgG2, IgG3, IgG4 (**A**), and IgM (**B**). IgG2 concentrations were higher in AD VI compared to Normal, and IgG4 levels were increased in AD III and AD VI. IgM levels were higher in AD V and AD VI (**B**). Data is shown as mean ± SEM. Overall difference was determined with Kruskal–Wallis test and pairwise comparisons were conducted with Wilcoxon rank sum tests. *p ≤ 0.05, **p ≤ 0.01 and ***p ≤ 0.001.

Native-state Ig isotypes in supernatants were quantified with Luminex Multi-Analyte Profiling technology. Using the Millipore Human Immunoglobulin Isotyping 5-plex assay, the presence and quantity of IgG1, IgG2, IgG3, IgG4, and IgM were measured (Kruskal–Wallis test : p = 0.436, p = 0.045, p = 0.366, p = 0.003, and p = 0.052, respectively) (Fig. 4A,B). IgM achieved a borderline significance in the overall comparison and further pairwise comparisons showed that IgM protein levels demonstrated a significant increase in AD V (p = 0.026) and AD VI (p = 0.029) compared to Normal. IgM levels between AD III and Normal were not significantly altered. IgG2 levels were significantly elevated in AD VI (p = 0.019) compared to Normal. In the analysis of IgG4, 2 out of 5 AD III and 4 out of 5 AD VI cases from Duke had concentrations above the range of detection, and one Normal and one AD V sample from NSW were below the range of detection. Due to limitations in quantification, the maximum (458.14 ng/mL) and minimum (0.031 ng/mL) measurable concentrations were assigned to cases that were out of range, with the understanding that the actual values are much greater or lower than presented. Under these limitations, IgG4 levels in AD III (p = 0.033) and AD VI (p = 0.003) were significantly elevated compared to Normal. However, levels of IgG4 in AD V were on par with Normal.

### Summary of Ig levels in AD compared to normal

The varying stages of AD were grouped together to determine the overall relationship between Ig’s and disease (Table 2). When evaluating the disease, IgG-γ, IgM-µ, IgG2, IgG4, IGHG2, IGHG4, IgG2 ng/mL, IgG4 ng/mL, and IgM ng/mL were demonstrated to be significantly elevated in AD (n = 16) compared to Normal (n = 9) (p = 0.023, p = 0.005, p = 0.008, p = 0.004, p = 0.002, p = 0.003, p = 0.02, p = 0.027, and p = 0.005, respectively).

**Table 2:** Each immunoglobin isotype and subclass quantified from cortical tissue supernatants was compared between cognitively normal individuals (Normal) and AD individuals (AD, all stages combined) using Wilcoxon rank sum tests.

Experiment	Full Sample (N = 25)	Normal (N = 9)	AD (N = 16)	P value
**IgG-gamma/actin, median (IQR)**	0.6 (0.3, 1)	0.3 (0.2, 0.6)	0.8 (0.6, 1.1)	**0.023**
**IgM-mu/actin, median (IQR**)	0.8 (0.6, 1.3)	0.6 (0.5, 0.6)	0.9 (0.7, 1.5)	**0.005**
IgG1/actin, median (IQR)	0.7 (0.4, 0.9)	0.4 (0.1, 0.9)	0.8 (0.5, 1)	0.276
**IgG2/actin, median (IQR)**	0.7 (0.3, 1.3)	0.1 (0, 0.4)	0.8 (0.6, 1.5)	**0.008**
IgG3/actin, median (IQR)	1 (0.6, 1.6)	0.8 (0.2, 1)	1.1 (0.8, 1.7)	0.187
**IgG4/actin, median (IQR)**	0.3 (0.1, 0.7)	0.1 (0, 0.2)	0.5 (0.2, 0.9)	**0.004**
Gene IGHG1, median (IQR)	4.9 (4.3, 5.6)	4.3 (4.1, 5.2)	5.1 (4.7, 5.8)	0.065
**Gene IGHG2, median (IQR)**	1.6 (0.7, 2)	0.5 (0.1, 1)	1.9 (1.6, 2.1)	**0.002**
Gene IGHG3, median (IQR)	1.4 (0.8, 2.2)	1.2 (0.2, 1.4)	1.7 (1.2, 2.2)	0.121
**Gene IGHG4, median (IQR)**	− 0.3 (− 1.2, 0.6)	− 2.4 (− 3.5, − 0.6)	0.3 (− 0.3, 0.7)	**0.003**
IgG1 ng/ml, median (IQR)	417.3 (303.5, 644.5)	330.7 (126.8, 573.4)	451.4 (342.1, 647.9)	0.246
**IgG2 ng/ml, median (IQR)**	304.8 (129.4, 455.7)	129.4 (86.4, 215.2)	392.9 (243.6, 602.2)	**0.020**
IgG3 ng/ml, median (IQR)	340.3 (62.5, 340.3)	178.8 (20.1, 340.3)	340.3 (318.2, 340.3)	0.093
**IgG4 ng/ml, median (IQR)**	42 (9.9, 349.4)	13.2 (4.9, 30.1)	159.5 (27.6, 458.1)	**0.027**
**IgM ng/ml, median (IQR)**	126.6 (46.7, 171.9)	34.6 (19.6, 91.2)	132.4 (118.2, 197.3)	**0.005**

Significantly altered levels are bolded. Data is represented as Mean, interquartile range [25th and 75th percentile].

## Discussion

The relationship between the immune system and the progression of AD has recently been investigated^12,13^. However, the association between various Ig isotypes and subclasses in AD parenchyma has not been evaluated. By examining fresh-frozen frontal cortex (FCx) brain tissue from pathologically diagnosed AD (Braak and Braak stage III, V, and VI) and age-matched cognitively normal individuals (Normal), we demonstrated that the levels of Ig’s were significantly altered in AD subjects compared to Normal. Furthermore, the trends of these Ig’s with increasing Braak and Braak stages suggest a relationship with the pathological progression of AD.

Immunoglobulin M (IgM) is the first antibody isotype produced by B-cells in an immune response that provides short term protection by opsonizing antigens/pathogens^30–35^. B-cells in the dura region of the meninges, either locally produced by the skull bone marrow or from the deep cervical lymph nodes, are genetically driven to produce IgM autoantibodies that assist in maintaining homeostasis^21,36^. Baulch and colleagues demonstrated a loss of B1 cells and their production of IgM-Aß, IgM autoantibodies targeting beta-amyloid (Aß) peptide 1–42, in the peripheral blood of 5XFAD mice compared to wild-type littermates^37^. Furthermore, the levels of IgM-Aß in peripheral blood of AD patients were also shown to be significantly lower than cognitively normal elderly individuals^15^. Our results demonstrate elevated levels of IgM in the FCx of late-stage AD (AD V and AD VI) compared to Normal, while IgM levels in early-stage AD (AD III) were comparable to Normal (Figs. 1B, 4B). Accumulation of proteins like Aß and phosphorylated Tau (p-Tau) in AD III is not as pronounced in the FCx as it is in the hippocampus, suggesting that at this pathological stage, an immunological response may not be warranted^5^. Increased protein accumulation in AD V FCx is accompanied with elevated IgM levels, which supports the idea that there may be a humoral immune response to AD pathology. This is compounded by the consistently elevated levels of IgM that are detected in AD VI FCx. If IgM detected in the FCx are autoantibodies produced to maintain neuronal health, then the continually elevated levels exhibited in late-stage AD suggest a potential dysregulation in immunological response, resulting in the accumulation of protein and neuronal atrophy.

Ig class switching is an important mechanism of an immune response that enables B-cells to switch isotype production from IgM to IgG, a higher affinity Ig that provides a tailored response against infection and long-term immunity^31,38^. Our results demonstrate elevated levels of IgG-γ in late-stage AD FCx, which supports the presence of an active and local humoral immune response in AD parenchyma (Fig. 1A), however, its relationship with disease severity remains undetermined. Kim and colleagues, using a 3xTgAD mouse model, demonstrated that an increase in activated B-cells in peripheral blood could infiltrate the parenchyma and increase pathology. In addition, the depletion of B-cells not only reduced the levels of Aß in the mouse brain tissue but also improved cognitive function, suggesting that cognitive impairment could be attributable to IgG and neuroinflammation^12^. Other researchers have demonstrated that removal of the humoral immune response in an AD transgenic mouse model accelerated AD pathology, which was ameliorated with administration of external IgG^13^. Furthermore, apoliporotein E (APOE) knock-in murine models demonstrated reduced levels of peripheral B-cells in APOE-4 knock in mice when compared to APOE-3, resulting in lower levels of total IgG in the brain of APOE-4 mice, suggesting a failure in immunological response^16^. These conflicting results may be dependent upon mouse models, IgG targets, or the IgG subclasses.

Investigations of AD FCx were expanded to examine the distribution of the 4 IgG subclasses, and the roles they may play in the humoral response to AD pathology. Using various techniques, we demonstrated a change in IgG subclass profile in AD FCx compared to Normal, with significant increases in the levels of IgG2 and IgG4 (Figs. 2, 3, 4). IgG-Aß autoantibodies, typically found in the sera of AD patients and elderly individuals, are predominantly IgG1 and IgG3 subclasses, and have been suggested to have beneficial effects on amyloid pathology and neurotoxicity due to their pro-inflammatory effector functions^39,40^. Therefore, if IgG1 and IgG3 in AD parenchyma are autoantibodies, then their unaltered levels may indicate a possible failure in the humoral response due to skewed presence of other IgG subclasses.

IgG2, the second most abundant IgG subclass in sera, was found to be elevated in late-stage AD parenchyma. While the relationship between IgG2 and neuronal disorders is unknown, it is suspected that the elevated levels may be related to increased IgG4 as suggested in other diseases^41–43^. IgG4 antibodies are generally formed in response to long-term antigen exposure in non-infectious situations and may become the dominant subclass^18^. IgG4 has a high affinity Fab region and has been shown to competitively bind to the target antigen to cause a “blocking” effect by preventing the binding of other effective Ig’s^18,19,44,45^. Thus, in cases of chronic exposure to antigen, IgG4 may suppress an immunological response^45^. Significantly elevated IgG4 antibodies presented in AD FCx could act as autoantibodies by binding to proteins, such as Aß and p-Tau, thereby blocking other immunologically active Ig from binding to those antigens. This could inhibit the natural process of debris clearance and accelerate abnormal protein buildup, resulting in neurodegeneration. This, however, may not be the only pathway in which IgG4 may be deleterious.

Analysis of IgG4 with Luminex technology, which quantifies native-state Ig’s by targeting the Fc region, demonstrated that the concentration of IgG4 in AD III subjects was significantly 4.8-fold higher compared to Normal (Fig. 4). This implies that IgG4 may play a role in the early stages of AD, prior to the toxic accumulation of Aß and p-Tau. Interestingly, the concentration of IgG4 in AD V subjects was similar to Normal, but increased by ninefold compared to Normal in AD VI. The drop in IgG4 concentration in AD V may be attributed to binding availability of the Fc region, since the levels were found to be consistently higher than normal when denaturing techniques were implemented. Additionally, the Fc region of IgG4 has been shown to bind to the Fc region of other Ig’s, inhibiting their effector functions^18,19,46–49^. This concept suggests that the Fc region of IgG4 in AD V FCx may be bound to a secondary Ig, obstructing an appropriate immunological response and consequently aiding the development of AD pathology.

It has been reported that a limited number of B-cells can reside in the parenchyma of healthy individuals, and their abundance increases with disease prevalence^36,50,51^. To explore whether local B-cells in the FCx were responsible for the observed Ig elevations, RNA was subjected to RT-qPCR. Detection frequency of IGHG2 and IGHG4 RNA transcripts in AD VI subjects were higher compared to Normal and earlier stages of disease (Table 1). This suggests that the IgG4 and IgG2 elevations exhibited in AD III and AD V may not be attributed to resident B-cells in the parenchyma but may be from meningeal B-cells or peripheral blood. In contrast, IGHG1 and IGHG3 RNA transcripts were detected among all groups evaluated. This suggests that local B-cells in AD parenchyma may be more inclined to produce IgG1 and IgG3 but not IgG2 and IgG4. The relationship between B-cells and AD needs further investigation to determine if isotype switching or IgG subclass production may be responsible for an improper immune response promoting neuroinflammation and disease pathogenesis.

While our results demonstrated elevations in IgG2, IgG4, and IgM levels in AD brain tissue, we recognize limitations of our investigations. First, the sample size of each test group was limited, and the absence of AD IV samples precluded a sequential and more thorough analysis that would encompass the various stages of AD progression. Second, examining brain tissues from subjects with Mild Cognitive Impairment would have provided further insight into neuroinflammatory events and offered another perspective on the roles of Ig’s and neurodegeneration in AD progression. Furthermore, in order to establish whether a distinct profile of Ig elevation is specific to AD, it would be beneficial to investigate other forms of neurodegenerative diseases to consider potential targets that are exclusive to AD.

This investigation presents the immune response as an upstream process to neuropathology in AD. Alteration in the Ig profile in AD parenchyma provides new insight into disease progression. Future investigations to clarify the targets of IgG4 and IgM may reveal their functions in AD pathogenesis, as well as opening new avenues in the study of neurodegeneration.

## Materials and methods

### Tissue samples

Fresh-frozen cortical tissue specimens from various pathologically diagnosed Braak and Braak stages of Alzheimer’s Disease (AD) and age-matched cognitively normal (Normal) brains were obtained from Kathleen Price Bryan Brain Bank of Duke University Medical Center, NC, USA, and from New South Wales (NSW) Tissue Resource Center at the University of Sydney and the Sydney Brain Bank at Neuroscience Research Australia, which are supported by the National Health and Medical Research Council of Australia, The University of New South Wales, Neuroscience Research Australia and Schizophrenia Research Institute. Research reported in this publication was supported by the National Institutes on Alcohol Abuse and Alcoholism of the National Institute of Health under Award number R28AA012725. The content is solely the responsibility of the authors and does not necessarily represent the official views of the National Institutes of Health. This study was approved by the institutional review board of Albert Einstein College of Medicine (AECOM), Bronx, NY vide number 2018-9204. The brain specimens were pathologically diagnosed by both banks using similar methods, which enabled their unification. Tissue samples consist of 9 Normal (age: 72–102, 7 female), 5 AD III (age: 72–89, 3 female), 6 AD V (age: 84–100, 5 female) and 5 AD VI (age: 68–84, 2 female). Details are presented in Supplementary Table 1.

### Tissue processing

Brain homogenates were processed by sonicating (Polytron) 1 g of fresh-frozen cortical gray matter devoid of meninges and visible blood vessels in 20 mL of lysis buffer [tris buffered saline, pH 7.4 with 1 mM sodium orthovanadate, 10 mM sodium fluoride, 2 mM EGTA, and 0.1% Triton-X-100] containing complete mini protease inhibitor cocktail EDTA-free (Roche). Homogenates were centrifugated at 14,000 RPM for 10 min at 4 °C (Eppendorf 5418R centrifuge) to remove insoluble particulates. Supernatant fractions were used for analysis. Protein concentrations were measured using the Bradford assay (BioRad Laboratories) and Nanodrop (ND1000 Thermo Fisher Scientific).

### Western Blot analysis

Supernatants were examined under denaturing conditions; 20 µg of protein were incubated with dithiothreitol (DTT) and heated at 100 °C for 5 min. Samples were loaded on to 10% Bis–Tris mini protein gels (NuPAGE, Invitrogen) and electrophoretic separation of proteins was achieved with NuPAGE MOPS SDS running buffer (Invitrogen). Proteins were transferred to PVDF membranes (EMD Millipore) in the Mini Blot Module (Invitrogen) with NuPAGE transfer buffer (Invitrogen) containing 7.5% methanol over the span of one hour. Membranes were blocked with 5% nonfat dry milk in PBS with 0.01% Tween-20 for one hour at room temperature in order to minimize non-specific binding. Subsequently, membranes were incubated over night at 4 °C with either horseradish peroxidase (HRP) conjugated rabbit anti-human IgG heavy chain (IgG-γ) (Abcam, 1:5000), HRP conjugated rabbit anti-human IgM heavy chain (IgM-µ) (Millipore; 1:4000), rabbit anti-human IgG1 (Abcam, 1:5000), rabbit anti-human IgG2 (Abcam, 1:5000), rabbit anti-human IgG3 (Abcam, 1:5000), or rabbit anti-human IgG4 (Abcam, 1:5000) primary antibodies. It is important to note that the antibodies against human IgG1-IgG4 have a strong binding affinity and require independent experimentation. Membranes undergoing IgG subclass protein detection were washed and incubated with HRP-conjugated anti-rabbit secondary antibody for 2 h at room temperature (Invitrogen, 1:5000). A full list of antibodies and their specificity can be found in supplementary Table 2. Protein bands were visualized with chemiluminescent reagent and KwikQuant imager as per manufactuer instructions (Kindle Bioscience). Densitometric analysis was performed with ImageJ software. Normalization using a loading control antibody was achieved by incubating membranes with mouse anti-Actin monoclonal antibody (Millipore, 1:5000) followed by goat anti-mouse IgM (Millipore 1:2000), or HRP-conjugated mouse anti-Beta Actin monoclonal antibody (Invitrogen, 1:2000). The HRP conjugated anti-Beta Actin prominent upper band was quantified as per manufactuers’ suggestion. To correct for technique variability, normalization was enabled by using two samples that were duplicated across the membranes, denoted with filled rhombus and ☨. Full length blots are provided in Supplementary Figs. 1 and 2.

### Proteomic analysis

Brain homogenates (100–300 µg) prepared as previously described were centrifugated at 13,000 RPM for 8 min at 4 °C (Sorvall Legend XTR centrifuge). The supernatants were collected, and reduced with 20 mM DTT at 56 °C for 30 min, followed by alkylation by subsequent addition of 40 mM iodoacetamide and a 20 min incubation in the dark. Trypsin digestion was performed using the S-trap digestion method. Briefly, 12% phosphoric acid was added at a 1.2% final concentration. Binding buffer (90% methanol/10 mM ammonium bicarbonate) was added in a 1:7 ratio and samples were loaded onto S-trap mini columns. These were washed three times with 90% methanol/10 mM ammonium bicarbonate. Trypsin (1:25 ratio) in 125 µL 50 mM ammonium bicarbonate, pH 8 was added onto the S-trap column and incubated overnight in a 37 °C water bath. The peptide digests were sequentially eluted from the S-trap column with 50 mM ammonium bicarbonate, 0.1% formic acid and 50% acetonitrile/0.1% formic acid and dried in a speed vacuum. The peptides were desalted by HLB C18 resin and eluted with 60% acetonitrile/0.1% Trifluoroacetic acid (TFA) and dried in a speed vacuum. The desalted peptides were resuspended in 0.1% TFA and analyzed by C18 RP-LC-ESI-MS/MS using the Ultimate 3000 nano-RSLC UPLC connected to the Lumos tribrid mass spectrometer. The resulting MS/MS data was processed by Proteome Discoverer v2.4. The protein database was searched against Swiss-Prot human protein database^52^. Label-free quantitation and statistical analysis by ANOVA was performed to determine the significant fold changes and p-value of identified proteins.

### Luminex multi-plex assay

Supernatant protein concentration of 30 µg per 25 µL lysis buffer were run in triplicate for all samples. Simultaneous quantification of IgG1-4 and IgM was performed using a Milliplex^®^ MAP Human Immunoglobulin Isotyping Magnetic Bead Panel Kit (#HGAMMAG-301K, EMD Millipore), according to the manufacturer’s instructions. The assay was read on a Luminex^®^ MAGPIX® analyzer with xPONENT^®^ software (Luminex Corporation) and the data was analyzed using a 5-parameter logistic curve on Milliplex Analyst Software (EMD Millipore).

### Quantitative reverse transcription PCR (RT-qPCR)

Quantitative Reverse Transcription PCR (RT-qPCR) for immunoglobulin heavy chain genes was performed at the Genomics Core Facility at AECOM, Bronx, NY, supported by the Cancer Center Support Grant (P30 CA013330). cDNA was prepared from 1 µg of total RNA using SuperScript™ VILO™ master mix (Invitrogen). RT-qPCR was performed using custom TaqMan assays designed for IGHG1-4 individual genes (Applied Biosystems) in 8 µL reaction volume containing TaqMan Fast Advanced Master Mix and TaqMan Primer Probe Mix as per manufacturer’s instructions and the 7900HT Fast Real-Time PCR instrument (supplementary Table 3). Eukaryotic 18S rRNA was used as the internal loading control (HS99999901_s1, Thermo Fisher Scientific). Fold-changes were calculated using the 2^−ΔΔCT^ method and presented as fold differences of relative gene expression normalized to human control RNA.

### Statistical analysis

First an overall comparison of the levels of each Ig class between the four experimental groups (Normal, AD III, AD V, and AD VI) was conducted using the Kruskal–Wallis test. If there was a significant overall difference, then pairwise comparisons were conducted using the Wilcoxon rank sum tests. To further evaluate AD as a whole regardless of stages, Ig levels were compared between grouped AD stages and Normal using Wilcoxon rank sum tests. All tests were two-sided and statistical significance was set as p < 0.05. All analyses were conducted using R 3.5.1^53^.

## Supplementary Information

Supplementary Information.
