# Supplementary material for: Altered abundances of human immunoglobulin M and immunoglobulin G subclasses in Alzheimer’s disease frontal cortex
Source: Sci Rep. 2022 Apr 28;12:6934. doi: 10.1038/s41598-022-10793-w (PMC9050688; doi:10.1038/s41598-022-10793-w)
Supplement: Supplementary file 1 — Supplementary Information. [file 41598_2022_10793_MOESM1_ESM.pptx]

## Slide 1
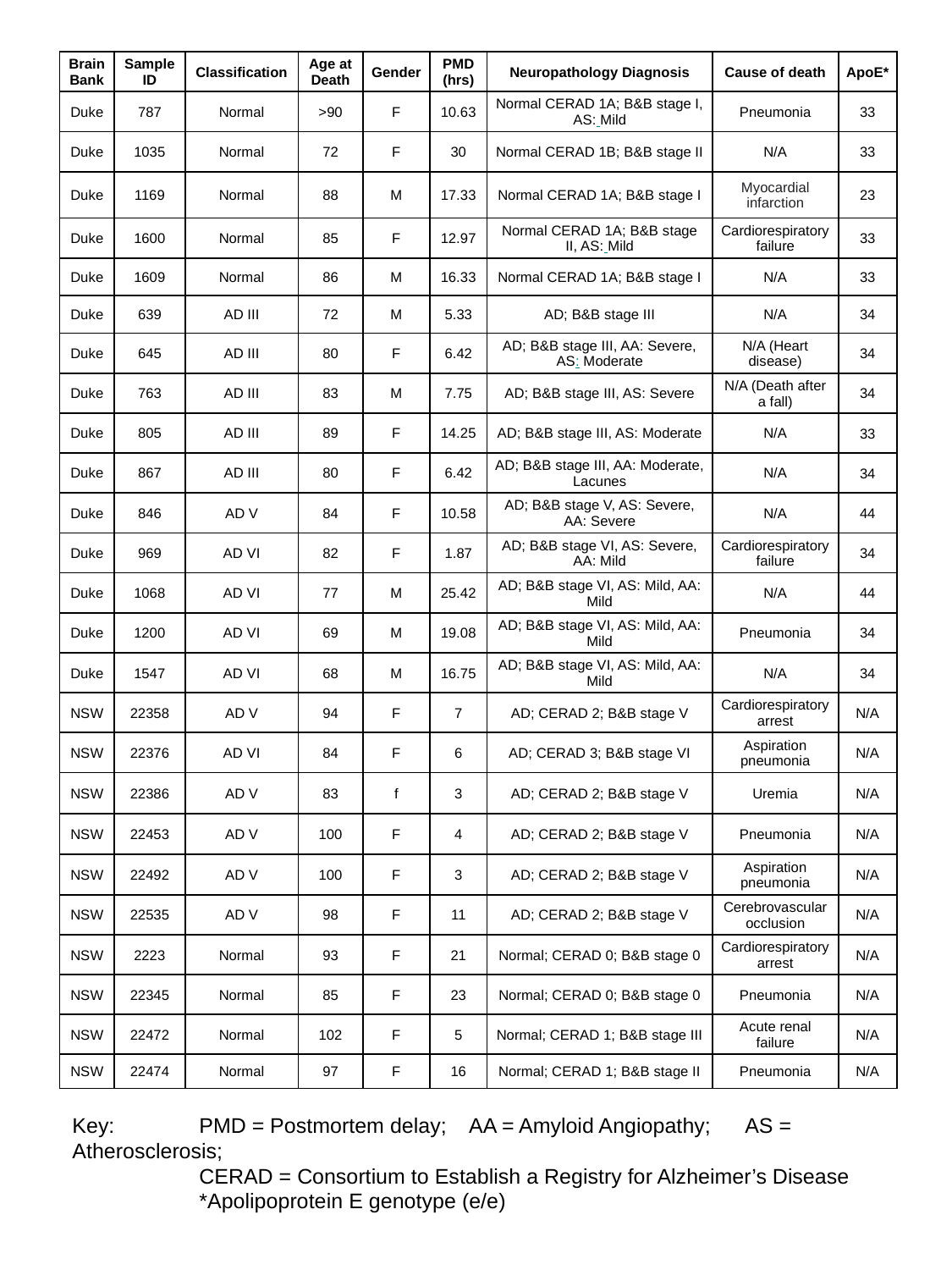

| Brain Bank | Sample ID | Classification | Age at Death | Gender | PMD (hrs) | Neuropathology Diagnosis | Cause of death | ApoE\* |
| --- | --- | --- | --- | --- | --- | --- | --- | --- |
| Duke | 787 | Normal | ˃90 | F | 10.63 | Normal CERAD 1A; B&B stage I, AS: Mild | Pneumonia | 33 |
| Duke | 1035 | Normal | 72 | F | 30 | Normal CERAD 1B; B&B stage II | N/A | 33 |
| Duke | 1169 | Normal | 88 | M | 17.33 | Normal CERAD 1A; B&B stage I | Myocardial infarction | 23 |
| Duke | 1600 | Normal | 85 | F | 12.97 | Normal CERAD 1A; B&B stage II, AS: Mild | Cardiorespiratory failure | 33 |
| Duke | 1609 | Normal | 86 | M | 16.33 | Normal CERAD 1A; B&B stage I | N/A | 33 |
| Duke | 639 | AD III | 72 | M | 5.33 | AD; B&B stage III | N/A | 34 |
| Duke | 645 | AD III | 80 | F | 6.42 | AD; B&B stage III, AA: Severe, AS: Moderate | N/A (Heart disease) | 34 |
| Duke | 763 | AD III | 83 | M | 7.75 | AD; B&B stage III, AS: Severe | N/A (Death after a fall) | 34 |
| Duke | 805 | AD III | 89 | F | 14.25 | AD; B&B stage III, AS: Moderate | N/A | 33 |
| Duke | 867 | AD III | 80 | F | 6.42 | AD; B&B stage III, AA: Moderate, Lacunes | N/A | 34 |
| Duke | 846 | AD V | 84 | F | 10.58 | AD; B&B stage V, AS: Severe, AA: Severe | N/A | 44 |
| Duke | 969 | AD VI | 82 | F | 1.87 | AD; B&B stage VI, AS: Severe, AA: Mild | Cardiorespiratory failure | 34 |
| Duke | 1068 | AD VI | 77 | M | 25.42 | AD; B&B stage VI, AS: Mild, AA: Mild | N/A | 44 |
| Duke | 1200 | AD VI | 69 | M | 19.08 | AD; B&B stage VI, AS: Mild, AA: Mild | Pneumonia | 34 |
| Duke | 1547 | AD VI | 68 | M | 16.75 | AD; B&B stage VI, AS: Mild, AA: Mild | N/A | 34 |
| NSW | 22358 | AD V | 94 | F | 7 | AD; CERAD 2; B&B stage V | Cardiorespiratory arrest | N/A |
| NSW | 22376 | AD VI | 84 | F | 6 | AD; CERAD 3; B&B stage VI | Aspiration pneumonia | N/A |
| NSW | 22386 | AD V | 83 | f | 3 | AD; CERAD 2; B&B stage V | Uremia | N/A |
| NSW | 22453 | AD V | 100 | F | 4 | AD; CERAD 2; B&B stage V | Pneumonia | N/A |
| NSW | 22492 | AD V | 100 | F | 3 | AD; CERAD 2; B&B stage V | Aspiration pneumonia | N/A |
| NSW | 22535 | AD V | 98 | F | 11 | AD; CERAD 2; B&B stage V | Cerebrovascular occlusion | N/A |
| NSW | 2223 | Normal | 93 | F | 21 | Normal; CERAD 0; B&B stage 0 | Cardiorespiratory arrest | N/A |
| NSW | 22345 | Normal | 85 | F | 23 | Normal; CERAD 0; B&B stage 0 | Pneumonia | N/A |
| NSW | 22472 | Normal | 102 | F | 5 | Normal; CERAD 1; B&B stage III | Acute renal failure | N/A |
| NSW | 22474 | Normal | 97 | F | 16 | Normal; CERAD 1; B&B stage II | Pneumonia | N/A |
Key: 	PMD = Postmortem delay; AA = Amyloid Angiopathy; AS = Atherosclerosis;
	CERAD = Consortium to Establish a Registry for Alzheimer’s Disease
	*Apolipoprotein E genotype (e/e)

## Slide 2
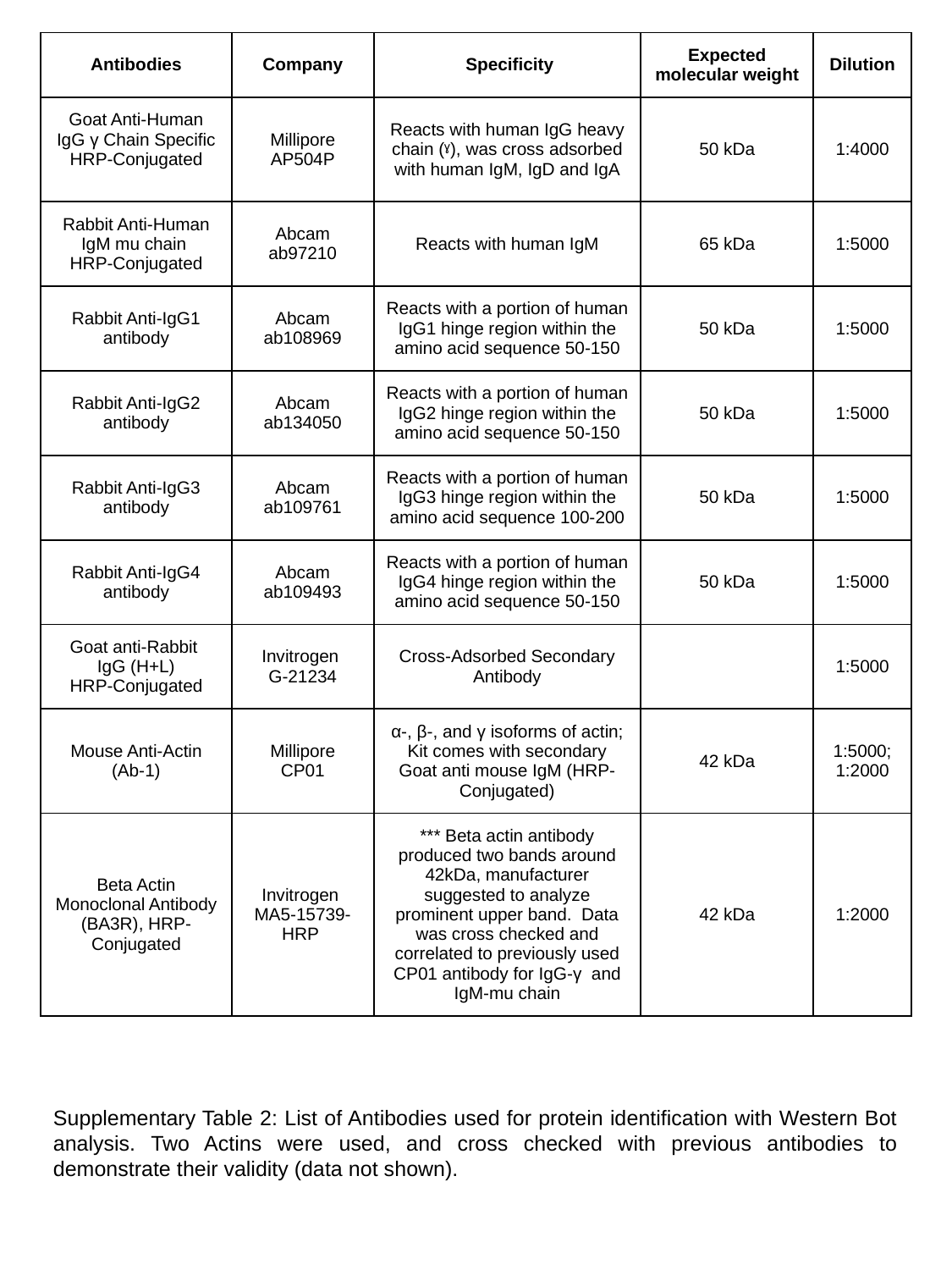

| Antibodies | Company | Specificity | Expected molecular weight | Dilution |
| --- | --- | --- | --- | --- |
| Goat Anti-Human IgG γ Chain Specific HRP-Conjugated | Millipore AP504P | Reacts with human IgG heavy chain (ˠ), was cross adsorbed with human IgM, IgD and IgA | 50 kDa | 1:4000 |
| Rabbit Anti-Human IgM mu chain HRP-Conjugated | Abcam ab97210 | Reacts with human IgM | 65 kDa | 1:5000 |
| Rabbit Anti-IgG1 antibody | Abcam ab108969 | Reacts with a portion of human IgG1 hinge region within the amino acid sequence 50-150 | 50 kDa | 1:5000 |
| Rabbit Anti-IgG2 antibody | Abcam ab134050 | Reacts with a portion of human IgG2 hinge region within the amino acid sequence 50-150 | 50 kDa | 1:5000 |
| Rabbit Anti-IgG3 antibody | Abcam ab109761 | Reacts with a portion of human IgG3 hinge region within the amino acid sequence 100-200 | 50 kDa | 1:5000 |
| Rabbit Anti-IgG4 antibody | Abcam ab109493 | Reacts with a portion of human IgG4 hinge region within the amino acid sequence 50-150 | 50 kDa | 1:5000 |
| Goat anti-Rabbit IgG (H+L) HRP-Conjugated | Invitrogen G-21234 | Cross-Adsorbed Secondary Antibody | | 1:5000 |
| Mouse Anti-Actin (Ab-1) | Millipore CP01 | α-, β-, and γ isoforms of actin; Kit comes with secondary Goat anti mouse IgM (HRP-Conjugated) | 42 kDa | 1:5000; 1:2000 |
| Beta Actin Monoclonal Antibody (BA3R), HRP-Conjugated | Invitrogen MA5-15739- HRP | \*\*\* Beta actin antibody produced two bands around 42kDa, manufacturer suggested to analyze prominent upper band. Data was cross checked and correlated to previously used CP01 antibody for IgG-γ and IgM-mu chain | 42 kDa | 1:2000 |
Supplementary Table 2: List of Antibodies used for protein identification with Western Bot analysis. Two Actins were used, and cross checked with previous antibodies to demonstrate their validity (data not shown).

## Slide 3
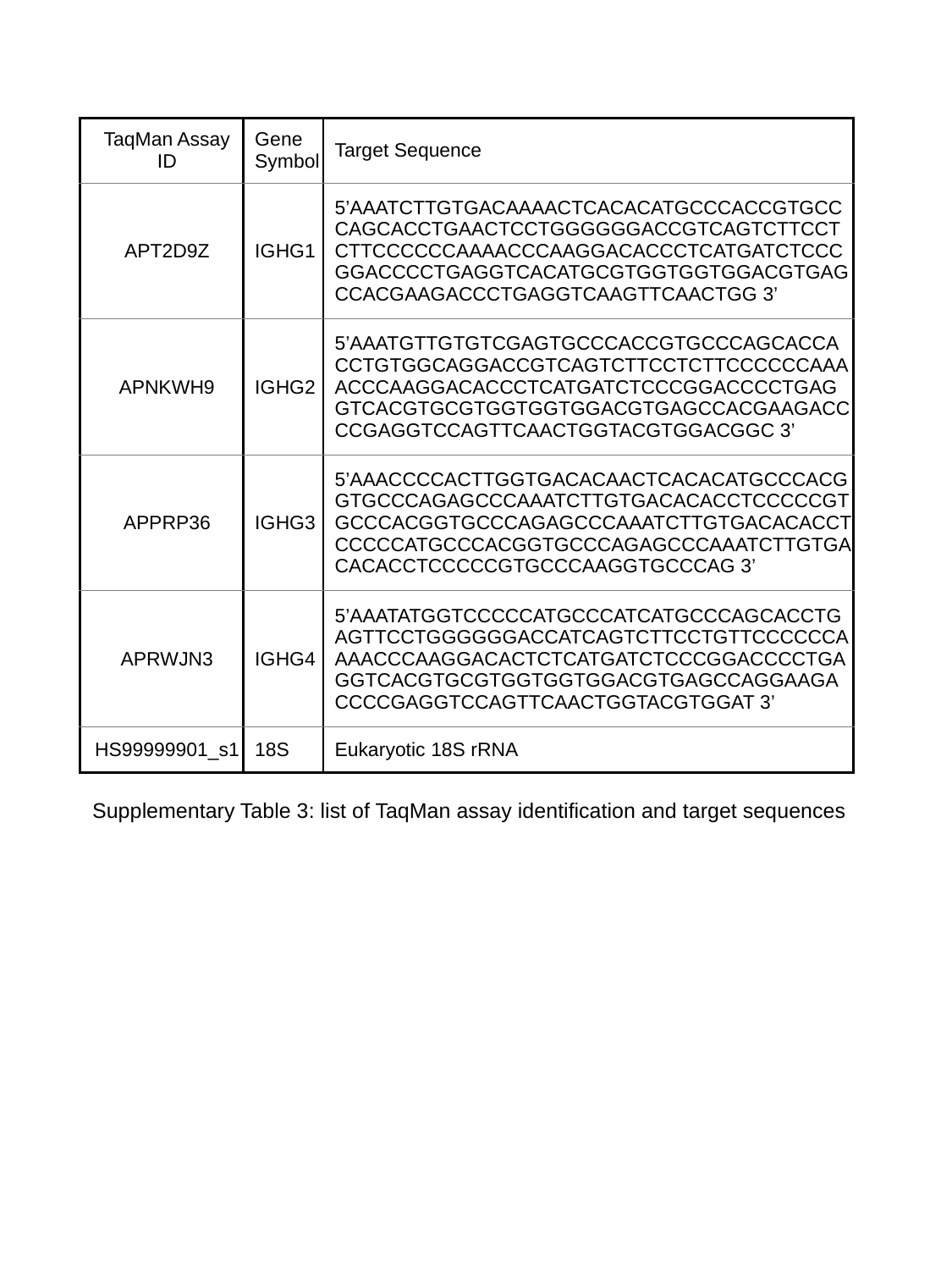

| TaqMan Assay ID | Gene Symbol | Target Sequence |
| --- | --- | --- |
| APT2D9Z | IGHG1 | 5’AAATCTTGTGACAAAACTCACACATGCCCACCGTGCCCAGCACCTGAACTCCTGGGGGGACCGTCAGTCTTCCTCTTCCCCCCAAAACCCAAGGACACCCTCATGATCTCCCGGACCCCTGAGGTCACATGCGTGGTGGTGGACGTGAGCCACGAAGACCCTGAGGTCAAGTTCAACTGG 3’ |
| APNKWH9 | IGHG2 | 5’AAATGTTGTGTCGAGTGCCCACCGTGCCCAGCACCACCTGTGGCAGGACCGTCAGTCTTCCTCTTCCCCCCAAAACCCAAGGACACCCTCATGATCTCCCGGACCCCTGAGGTCACGTGCGTGGTGGTGGACGTGAGCCACGAAGACCCCGAGGTCCAGTTCAACTGGTACGTGGACGGC 3’ |
| APPRP36 | IGHG3 | 5’AAACCCCACTTGGTGACACAACTCACACATGCCCACGGTGCCCAGAGCCCAAATCTTGTGACACACCTCCCCCGTGCCCACGGTGCCCAGAGCCCAAATCTTGTGACACACCTCCCCCATGCCCACGGTGCCCAGAGCCCAAATCTTGTGACACACCTCCCCCGTGCCCAAGGTGCCCAG 3’ |
| APRWJN3 | IGHG4 | 5’AAATATGGTCCCCCATGCCCATCATGCCCAGCACCTGAGTTCCTGGGGGGACCATCAGTCTTCCTGTTCCCCCCAAAACCCAAGGACACTCTCATGATCTCCCGGACCCCTGAGGTCACGTGCGTGGTGGTGGACGTGAGCCAGGAAGACCCCGAGGTCCAGTTCAACTGGTACGTGGAT 3’ |
| HS99999901\_s1 | 18S | Eukaryotic 18S rRNA |
Supplementary Table 3: list of TaqMan assay identification and target sequences

## Slide 4
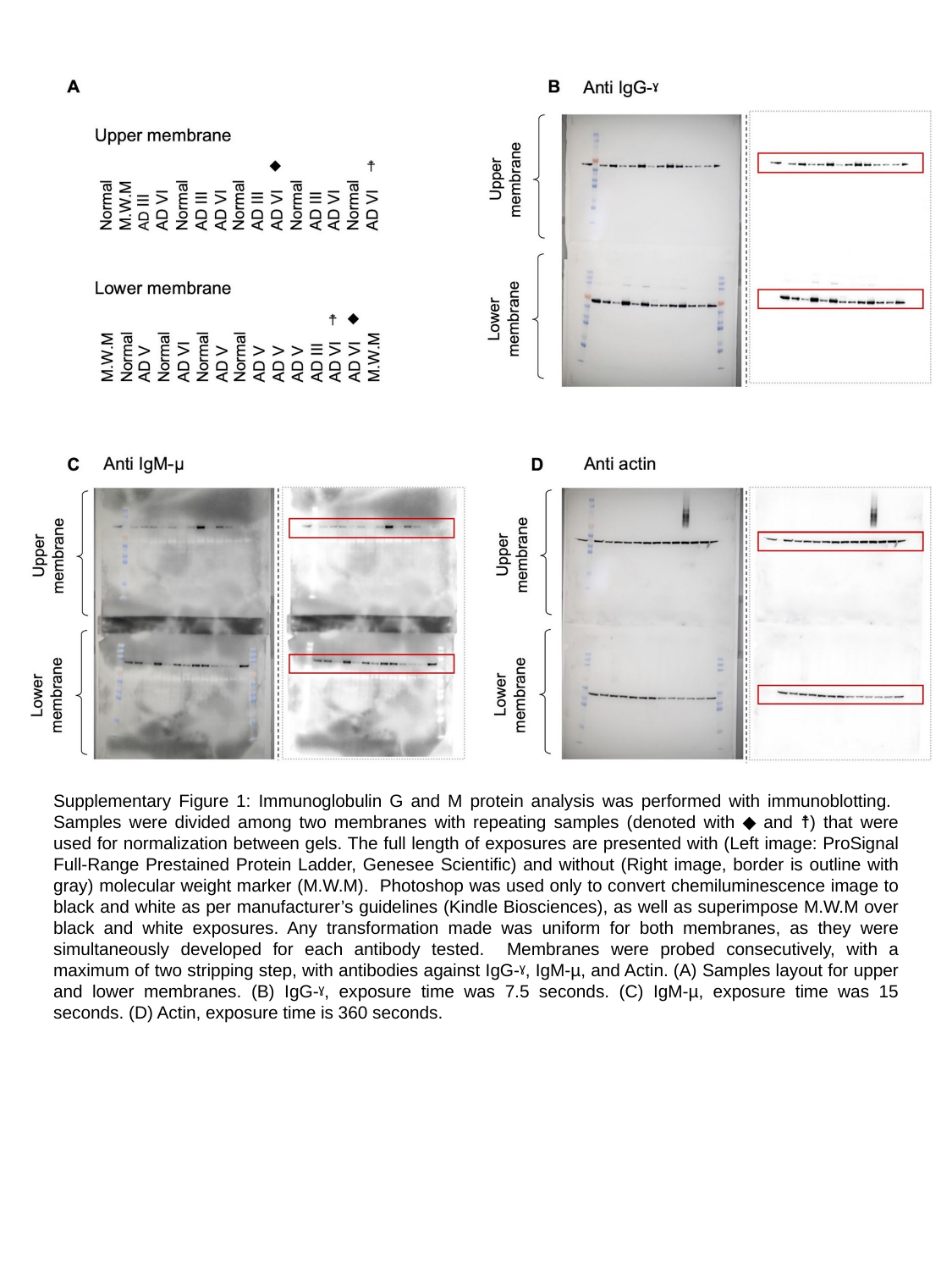

Supplementary Figure 1: Immunoglobulin G and M protein analysis was performed with immunoblotting. Samples were divided among two membranes with repeating samples (denoted with ◆ and ☨) that were used for normalization between gels. The full length of exposures are presented with (Left image: ProSignal Full-Range Prestained Protein Ladder, Genesee Scientific) and without (Right image, border is outline with gray) molecular weight marker (M.W.M). Photoshop was used only to convert chemiluminescence image to black and white as per manufacturer’s guidelines (Kindle Biosciences), as well as superimpose M.W.M over black and white exposures. Any transformation made was uniform for both membranes, as they were simultaneously developed for each antibody tested. Membranes were probed consecutively, with a maximum of two stripping step, with antibodies against IgG-ˠ, IgM-µ, and Actin. (A) Samples layout for upper and lower membranes. (B) IgG-ˠ, exposure time was 7.5 seconds. (C) IgM-µ, exposure time was 15 seconds. (D) Actin, exposure time is 360 seconds.

## Slide 5
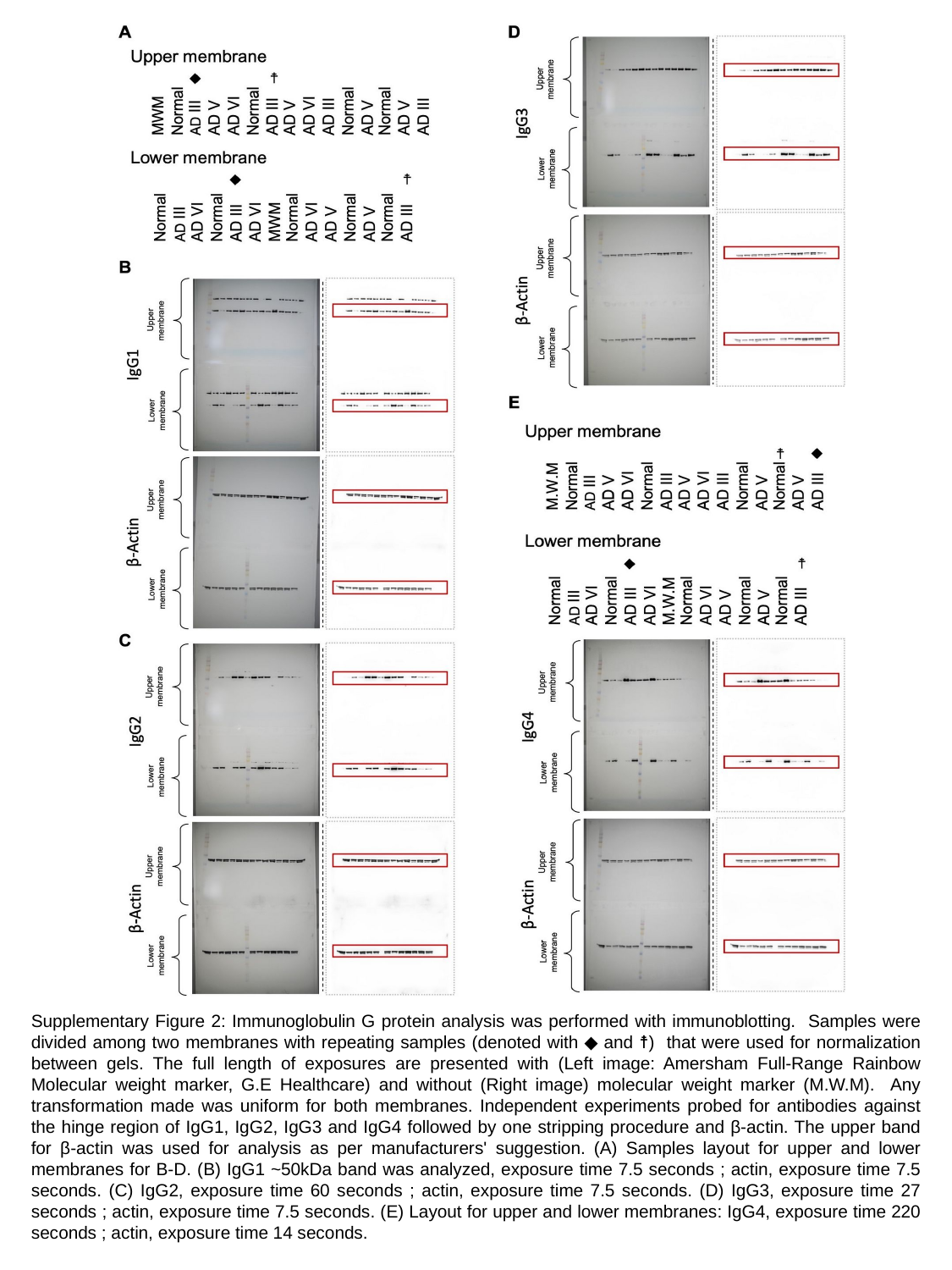

Supplementary Figure 2: Immunoglobulin G protein analysis was performed with immunoblotting. Samples were divided among two membranes with repeating samples (denoted with ◆ and ☨) that were used for normalization between gels. The full length of exposures are presented with (Left image: Amersham Full-Range Rainbow Molecular weight marker, G.E Healthcare) and without (Right image) molecular weight marker (M.W.M). Any transformation made was uniform for both membranes. Independent experiments probed for antibodies against the hinge region of IgG1, IgG2, IgG3 and IgG4 followed by one stripping procedure and β-actin. The upper band for β-actin was used for analysis as per manufacturers' suggestion. (A) Samples layout for upper and lower membranes for B-D. (B) IgG1 ~50kDa band was analyzed, exposure time 7.5 seconds ; actin, exposure time 7.5 seconds. (C) IgG2, exposure time 60 seconds ; actin, exposure time 7.5 seconds. (D) IgG3, exposure time 27 seconds ; actin, exposure time 7.5 seconds. (E) Layout for upper and lower membranes: IgG4, exposure time 220 seconds ; actin, exposure time 14 seconds.
